# Supplementary material for: Integrative GWAS and RNA-Seq analysis for target identification and virtual drug screening in colorectal cancer
Source: PLoS One. 2025 Oct 27;20(10):e0333179. doi: 10.1371/journal.pone.0333179 (PMC12558462; doi:10.1371/journal.pone.0333179)
Supplement: Attachment — Submitted filename: TO THE EDITORS.pdf [file pone.0333179.s003.pdf]

University of Baghdad/
College of Science for Women
Biology department
Baghdad
14 /6 /2025

Dear Editors in PLOS ONE

I hope you are fine.

Thank you to give me the opportunity to review the manuscript entitled (Integrative GWAS and RNA-Seq Analysis for Target Identification and Virtual Drug Screening in Colorectal Cancer).

The study focuses on the wide genomic data analysis of several SNPs associated with colorectal cancer, in the same time it suggested a potential therapeutic drug that targeted the expression of specific genes specially, PYGL, which may important role in colorectal cancer treatment and cure.

Its noteworthy, this article, may lead to applied the suggested targeted therapies in experimental or clinical trial after subjected it to validation as well as further laboratory evaluations and adjustments. Furthermore, other limitation may the restriction of the study to the Asian population, which may not align with the genetic predispositions in other populations.

Best regards,

Luma Al Obaidy
