# Supplementary material for: Integrative GWAS and RNA-Seq analysis for target identification and virtual drug screening in colorectal cancer
Source: PLoS One. 2025 Oct 27;20(10):e0333179. doi: 10.1371/journal.pone.0333179 (PMC12558462; doi:10.1371/journal.pone.0333179)
Supplement: Attachment — Submitted filename: Response to Reviewers.docx [file pone.0333179.s005.docx]

**Response to Reviewers**

**Dear Editor and Reviewers,**

Thank you very much for the opportunity to revise our manuscript entitled “Integrative GWAS and RNA-Seq Analysis for Target Identification and Virtual Drug Screening in Colorectal Cancer” (Manuscript ID: PONE-D-25-19455R1).

We sincerely appreciate the time and expertise you dedicated to evaluating our work. The constructive comments have been invaluable in refining our research, and we have made extensive revisions accordingly.

In the revised version, changes are highlighted in red, and our responses to each comment are detailed below.

Reviewer #1: Summary: This study attempts to find potential treatment targets and drug candidates for colorectal cancer by combining previously published genome-wide association studies (GWAS) with gene expression data (RNA-Seq) and drug screening through computational docking. The authors use existing public data to identify genes commonly linked to colorectal cancer in East Asian populations and suggest a few of them, such as PYGL and SMAD7, as potential targets. They then use molecular docking to screen for drugs that might bind to these targets. The results suggest a list of small molecules that could theoretically be repurposed to treat colorectal cancer. However, all analyses were performed with previously published datasets, and there was no experimental validation.

Strengths:

1. The manuscript is clearly written and well-structured, though excessively long for the novelty it presents.

2. Figures and tables are presented with clarity, though mostly re-confirm well-established findings.

Major Comments:

1. Lack of Novelty in Data or Methodology: The manuscript relies entirely on publicly available GWAS (e.g., bbj-a-76, GCST009869) and RNA-Seq (TCGA-COAD, TCGA-READ) datasets. No new data generation or significant methodological innovation is evident.

**Response: We selected a new GWAS data, and SNPs with 5e-8 < p-value < 1e-6 were included in the analysis.**

2. Absence of Experimental Validation

**Response: We greatly appreciate your suggestion to strengthen the conclusions through experimental verification. However, as this study is preliminary and exploratory in nature, we are currently unable to perform cellular and animal experiments. We have noted this limitation in the Discussion section and intend to conduct relevant cellular and animal experiments in our next study.**

3. Overstated Claims: The manuscript claims to offer a "theoretical framework for drug repurposing strategies" and makes repeated references to “promising therapeutic targets,” yet all results are entirely in silico, and the novelty is limited.

**Response: Revised.**

4. Redundancy With Existing Literature: The identified targets (e.g., SMAD7, TCF7L2, PYGL) have been widely studied in colorectal cancer, and their roles are well-documented. The manuscript does not provide fresh insights into these genes functions or offer a new angle on their clinical relevance.

**Response: In this study, GWAS data with p < 1e-6 were used as the initial screening criterion, identifying genes such as CDKN2B, BOC, and METRNL as having potential research value in colorectal cancer. These findings may also be applicable to studies of other diseases, providing valuable insights.**

Reviewer #2: This manuscript presents results from a workflow that integrates GWAS and RNA-seq analyses for identifying risk genes in colorectal cancer (CRC) and conduct targeted virtual drug screening. The authors report on 13 potential risk genes, with the PYGL representing the most promising therapeutic target, for which ten small molecule inhibitors were identified as potential drug targets for CRC.

The following are my major comments:

(1) A significant portion of the Introduction discusses Mendelian Randomization (MR), a method for investigating the causal relationships between exposures and some outcome, typically disease phenotypes. And further, the analysis of archived GWAS data was done in part using the R package TwoSampleMR, with the aim of minimizing weak instrumental variables, a key step in any MR analysis. And yet, curiously, no MR analysis was actually performed in this study. This needs clarification.

**Response: The TwoSampleMR package was used in this study, but no Mendelian Randomization (MR) analysis was performed; thus, it has been removed.**

(2) For the GWAS data analysis, the summary level data from three GWAS datasets were examined. Simply, SNPs with P-values less than 1E-5 and r^2 less than 0.001 within a large 10,000 kb (i.e., 10 Mb) sliding window were retained. Presumably there is a substantial overlap in the genome-wide SNPs tested in these datasets. If so, most of the SNPs are likely to have multiple association P-values (i.e., from each of three GWAS datasets). How was this handled? In the Results, a Manhattan plot is presented (Figure 2), but for only one of the three GWAS datasets (bbj-1-107). Further, the SNPs that passed the filtering thresholds are discussed per dataset, giving the reader the impression that each GWAS dataset was indeed examined examined separately, with each batch of SNPs simply lumped together to comprise the final list. If so, this is problematic. Typically some sort of meta-analysis is performed to combine evidence across the three datasets (as opposed to disregarding data and cherry-picking the best result per SNP). Common methods include inverse-variance weighted fixed-effects meta-analysis or random-effects meta-analysis (e.g., METAL, GWAMA). This will yield a single P-value and effect estimate per SNP across the three datasets. Please discuss.

**Response: The GCST90018808 dataset was selected, with p < 1e-6 as the screening threshold (Chen et al.2024).**

**Ref: Zhishan Chen, Xingyi Guo, Ran Tao, Jeroen R. Huyghe, Philip J. Law, Ceres Fernandez-Rozadilla, and et al. Fine-mapping analysis including over 254,000 East Asian and European descendants identifies 136 putative colorectal cancer susceptibility genes. *Nature Communications*. 2024,15: 3557.**

(3) From the GWAS analysis, 27 genes "significantly associated with CRC" were identified (NOTE: in Figure 1, this is listed as 28 genes). Of these, 13 genes (including lncRNAs) show significant differential expression in colon cancer tissue (NOTE: gene expression results for rectal cancer (READ) are not provided, despite being analyzed separately). Although each of these 13 genes harbor at least one significant GWAS SNP related to CRC, the relationship of these SNPs to cis gene expression was not examined (i.e., eQTLs). SNPs can have disease risk effects that stem from deleterious changes to an encoded protein impacting function (e.g., nonsynonymous mutations), with *no* effect on gene expression levels. Thus, going from a SNP association with CRC to differential gene expression in CRC without actually examining the relationship between the SNP and gene expression to link the two appears somewhat problematic. Please discuss.

**Response: In Figure 1, 28 genes were listed, but the correct number should be 27—this was an error. The associations between SNPs and genes in this study can be retrieved from published literature (S1 Table), so eQTL analysis data were not explicitly listed.**

(4) For the enrichment analysis for GO terms and KEGG pathways, it was performed on a list of only 13 genes. Except in cases of exceptional enrichment, this list is too small to yield anything informative. Looking at the top results (Tables 3 and 4), most of the GO terms and pathways involve only a single gene in the list (with a few having two genes). This doesn't provide any real insight. The point of such tests is to find wider patterns that connect various genes in their function and/or biological processes and represent an overrepresentation of such characteristics. Finding a single gene from the 13 genes that is also among the genes involved in, say, "starch and sucrose metabolism" doesn't tell you much, despite an adjusted P-value less than 0.1. This type of gene annotation can be achieved using various databases without resorting to enrichment testing. The authors may want to reconsider how to conduct this step in their study design.

**Response: A total of 24 significantly differentially expressed genes were identified, of which 17 mRNAs were selected for GO term and KEGG pathway analysis.**

(5) The gene PYGL was selected for "virtual screening via molecular docking analysis". Why this gene was selected from the list of 13 genes is not clear to this reviewer. Please explain.

**Response:Section 1.8 outlines the gene screening methodology.**

**“Structural data were obtained from the AlphaFold Protein Structure Database and the Protein Data Bank (PDB), with priority given to PDB entries. Selection criteria included the scientific name of the source organism (Homo sapiens), experimental method (X-ray diffraction or electron microscopy), and a refinement resolution of ≤2.5 Å.**

**A total of 17 mRNAs were identified through screening, among which only 3 possessed PDB IDs and contained small-molecule ligands. Based on the properties and size of the active sites, PYGL was ultimately selected for further investigation.**

Table 1. Structural data of the 17 mRNA-encoded proteins

| No. | Gene symbol | RNA type | PDB | | AlphaFold |
| --- | --- | --- | --- | --- | --- |
|  |  |  | PDB ID | Ligand ID |  |
| 1 | CDKN2B | mRNA |  |  | P42772 |
| 2 | BOC | mRNA | 3N1G | / |  |
| 3 | SCG5 | mRNA |  |  | P05408 |
| 4 | LRP1 | mRNA | 1D2L | / |  |
| 5 | ZCWPW2 | mRNA | 4Z0R | EDO: C_2_H_6_O_2_ |  |
| 6 | PYGL | mRNA | 3DDS | 26B: C_29_H_35_N_3_O_5_ |  |
| 7 | PREX1 | mRNA | 6VSK | / |  |
| 8 | SMAD7 | mRNA | 7CD1 | / |  |
| 9 | SLCO2A1 | mRNA |  |  | Q92959 |
| 10 | METRNL | mRNA |  |  | Q641Q3 |
| 11 | POU5F1B | mRNA |  |  | Q06416 |
| 12 | ABHD12B | mRNA |  |  | Q7Z5M8 |
| 13 | TCF7L2 | mRNA | 1JDH | / |  |
| 14 | CDH3 | mRNA | 5JYL | GOL: C_3_H_8_O_3_ |  |
| 15 | SOX6 | mRNA |  |  | P35712 |
| 16 | NKX2-3 | mRNA |  |  | Q8TAU0 |
| 17 | TMEM238L | mRNA |  |  | A6NJY4 |

Reviewer #3: This manuscript integrates genome-wide association study (GWAS) data from East Asian populations with RNA-Seq data from TCGA to identify colorectal cancer (CRC)-related genes, followed by virtual drug screening using molecular docking. The study highlights several candidate targets including PYGL, SMAD7, and TCF7L2, and proposes ten small-molecule compounds with potential therapeutic effects.

While the concept of integrating multi-omics data for drug discovery is promising and timely, the manuscript has several methodological and interpretational weaknesses that need to be addressed before it can be considered for publication.

Major Concerns

Despite the mention of Mendelian Randomization and LD-based SNP filtering, there is no actual demonstration of SNP-gene expression links through eQTL analysis. Without this, the integration between GWAS and RNA-Seq remains superficial. eQTL or TWAS analyses are essential to strengthen the causal inference between SNPs and gene expression in CRC.

Key genes (e.g., PYGL, SMAD7, TCF7L2) are listed, but their roles in CRC progression are not deeply discussed. More mechanistic insight or network analysis is needed.

The manuscript requires significant language editing for clarity and scientific tone. Several sections are repetitive or loosely organized.

Moreover, the manuscript lacks a clear and concise conclusion that synthesizes the key findings and their biological or clinical implications. A strong closing section is needed to articulate the overall contribution of the study.

Minor Issues

GWAS results need QQplot.

The format of table is not consisted.

Limitations should be stated more explicitly

**Response: The associations between SNPs and genes in this study are supported by published literature (S1 Table), providing indirect evidence for SNP-gene expression correlations. Therefore, eQTL analysis data were not explicitly presented.**

**Language editing were made to enhance scientific tone, including the removal of redundant content. The Conclusions section was expanded to better emphasize the key findings of this study.**

Reviewer #4: In this paper, the authors systematically identified key genes and potential drug targets associated with colorectal cancer in East Asian populations by integrating GWAS and RNA-Seq data, combined with molecular docking techniques. The authors also performed differential expression analysis on RNA-Seq data from TCGA to refine the findings. Overall, the paper is well-written. Below are my comments on the paper.

• In 1.2 Data Acquisition, the author mentioned that “Datasets were filtered based on case-control status and sample size, with smaller sample sizes excluded to ensure statistical robustness.” Could the author be more specific on how they determine if the sample size of a dataset is small.

**Response: Additional content has been included, with sample sizes (ncase) < 5000.**

• It would be better if the authors could briefly introduce the three GWAS dataset used. Are there any population substructure in each of the dataset?

**Response:**

| GWAS ID | Case (n) | Control (n) | Number of SNPs | Population | Ref. |
| --- | --- | --- | --- | --- | --- |
| GCST90018808 | 6,581 | 463,421 | 25,843,452 | European | [10] |
|  | 8,305 | 159,386 |  | East Asian |  |

• It seems to me that the authors are more interested in identifying CRC-related genes. If this is the case, what about using set-based genetic association analysis (e.g. SKAT by Wu et al. (2011)) directly to identifying disease-related genes? Are there any advantages of using SNP-based GWAS compared to set-based GWAS in your study?

Ref: Wu MC, Lee S, Cai T, Li Y, Boehnke M, Lin X. Rare-variant association testing for sequencing data with the sequence kernel association test. Am J Hum Genet. 2011 Jul 15;89(1):82-93. doi: 10.1016/j.ajhg.2011.05.029. Epub 2011 Jul 7. PMID: 21737059; PMCID: PMC3135811.

**Response: SKAT is a regression-based method, it can be easily extended to survival, and longitudinal and multivariate phenotypes. Both SNP-based GWAS and set-based GWAS are viable approaches for GWAS studies. However, set-based GWAS has received less attention in our current work, and further investigations in this direction are planned for future research.**

Reviewer #5: General Comments:

This study attempts an integrative approach combining GWAS and RNA-Seq data with molecular docking to identify therapeutic targets and drug candidates for colorectal cancer. The authors aim to bridge genetic insights with drug discovery, which is a valuable objective. However, the current methodology and presentation have several limitations that significantly impact the robustness and reliability of the findings. The conclusions drawn appear to be overstated given the foundational weaknesses.

Specific Comments:

1. The study uses three separate GWAS datasets. Given this, a formal meta-analysis of the GWAS data would significantly increase statistical power and the robustness of identified SNPs and genes. Alternatively, only retaining consistently replicated significant findings across at least two datasets could enhance accuracy and reduce false positives.

**Response: This study utilized one GWAS dataset. Differentially expressed genes were initially identified through GWAS screening and subsequently validated using RNA-seq data to reduce the probability of false positives.**

2. The significance threshold for SNP selection (p<1e−5) is relatively loose for GWAS, especially without stringent meta-analysis or replication across multiple independent cohorts. This increases the risk of including false-positive associations. Please justify this threshold more thoroughly or consider a stricter criterion.

**Response: In this study, a threshold of p < 1e-6 was applied for initial GWAS screening to identify differentially expressed genes, which were then confirmed through RNA-seq analysis to minimize false positive results.**

3. The inclusion criteria for GWAS datasets mentions filtering based on "smaller sample sizes excluded to ensure statistical robustness". However, specific thresholds for exclusion are not provided. Please clarify what constitutes a "smaller sample size" in this context.

**Response: Additional content has been included,, with sample sizes (ncase) < 5000.**

4. The linkage disequilibrium (LD) filtering criteria (r2<0.001 and a window size of 10,000 kb) seem unusually broad, potentially leading to very few independent SNPs or a lack of fine-mapping resolution. Please provide a more detailed justification for these specific parameters and their implications for instrumental variable selection.

**Response: The parameters were reconfigured with an r² < 0.1 and a window size of 100,000 kb."**

5. The methods state that "genes and samples with more than 50% missing (NA) values were removed." This is a very lenient threshold, and keeping data with nearly 50% missingness, especially when followed by imputation, raises concerns about data quality and potential biases introduced by the imputation process. Please justify why such a high percentage of missing values was tolerated and discuss its potential impact.

**Response: No missing values (NA) were present in the dataset. For data conversion using Sangerbox, the thresholds for row and column exclusion were set at 1% missing values."**

6. The fold-change threshold of 2.0 (equivalent to |logFC|>1.0) for differential expression is not quite strict. Consider if the current stringency is appropriate for the downstream analyses.

**Response: These genes were identified through GWAS analysis, followed by subsequent survival analysis, GO, and KEGG analyses. A fold-change threshold of 2.0 was employed as the standard criterion.**

7. Colorectal cancer incidence and mortality rates are noted to differ between males and females. A sex-stratified analysis could reveal important biological differences and sex-specific therapeutic targets. Please consider performing such an analysis or discussing its implications as a limitation and future direction.

**Response: Gender-stratified survival analysis revealed differential gene expression patterns between males and females. Due to the substantial volume of gender-specific data generated, these results are not presented in this study but will be addressed in future research."**

**Male**

**Female**

Fig. 1 The overall survival (OS) analysis of the *PYGL* gene in colorectal cancer.

8. The results show very few genes being shared across the three GWAS datasets. This lack of strong replication across datasets is a concern. The authors should explicitly discuss potential reasons for this limited overlap (e.g., population-specific effects, heterogeneity in study design, statistical power differences, or lack of truly robust signals) and its implications for the generalizability of the findings.

**Response: This study employed one GWAS dataset."**

9. The GO and KEGG enrichment analyses appear weak, with many terms including only one or two genes. This makes it difficult to draw strong biological conclusions. The background gene set used for the enrichment analysis should be clearly indicated. Were all genes in the human genome used as background, or a more relevant subset (e.g., all genes expressed in colon/rectal tissue)? The adjusted p-values for many terms in Table 3 and Table 4 are relatively high, suggesting marginal significance despite being "top 10." The conclusions drawn from these enrichment results should be very carefully tempered.

**Response: Additional content has been incorporated, with all genes in the human genome serving as the background reference.**

10. Please clarify why adjusted p-values were not used for the online drug screening results. Unadjusted p-values in such a large-scale screening can lead to a high number of false positives.

**Response: Adjusted p-values have been included in the analysis."**

11. The gene PYGL is highlighted as a key finding, but no validation is presented using external data or wet-lab experiments.

**Response: Survival analysis was performed for *PYGL*.**

12. The most significant limitation of this study is the complete lack of experimental validation for the identified therapeutic targets and the candidate small-molecule inhibitors. The findings are entirely computational, and without in vitro or in vivo validation, their therapeutic potential remains speculative. This should be explicitly stated as a major limitation in the discussion, with a clear commitment to future experimental work. The authors mention "the incorporation of extensive positive and control datasets enhances the robustness and reliability of the findings" in the discussion of limitations. While this is a good practice for computational studies, it does not substitute for independent experimental validation. This statement should be rephrased to avoid overstating the current study's robustness in a biological context.

**Response: Additional descriptions have been added: “evaluating the therapeutic efficacy of the selected genes and compounds using *in vitro* cellular assays and *in vivo* animal models.”**

13. The supplementary table file names do not consistently match the table numbers inside the files.

**Response: Revised**

Reviewer #6: To the Author,

Thanks for this valuable work.

Its noteworthy, this article, may lead to applied the suggested targeted therapies in experimental or clinical trial after subjected it to validation as well as further laboratory evaluations and adjustments. Furthermore, other limitation may the restriction of the study to the Asian population, which may not align with the genetic predispositions in other populations.

**Response: Thanks, The study population consisted of European and East Asian.**
