# Supplementary material for: Integrative GWAS and RNA-Seq analysis for target identification and virtual drug screening in colorectal cancer
Source: PLoS One. 2025 Oct 27;20(10):e0333179. doi: 10.1371/journal.pone.0333179 (PMC12558462; doi:10.1371/journal.pone.0333179)
Supplement: Attachment — Submitted filename: Response to Reviewers 20250815.docx [file pone.0333179.s006.docx]

**Response to Reviewers**

**Dear Editor and Reviewers,**

Thank you very much for the opportunity to revise our manuscript entitled “Integrative GWAS and RNA-Seq Analysis for Target Identification and Virtual Drug Screening in Colorectal Cancer” (Manuscript ID: PONE-D-25-19455R1).

We sincerely appreciate the time and expertise you dedicated to evaluating our work. The constructive comments have been invaluable in refining our research, and we have made extensive revisions accordingly.

In the revised version, changes are highlighted in red, and our responses to each comment are detailed below.

Reviewer #1: The prior comments still hold true regarding the lack of novelty in data or methodology for the analysis and results presented in the study.

**Response: Thank you for your time and expertise.**

Reviewer #3: This manuscript presents a promising integrative in silico approach combining GWAS, RNA-Seq, and molecular docking to identify therapeutic targets for colorectal cancer. The authors have addressed many of the reviewers' previous concerns, including data selection, method transparency, and overstatement of claims. However, several key issues remain unresolved:
The lack of direct SNP-expression linkage (e.g., eQTL or TWAS) weakens the connection between genetic association and expression.
nsufficient explanation for selecting PYGL for molecular docking over other candidates (e.g., SMAD7, TCF7L2).

Paper foamting and Language still to be polished.
Still want have QQ plot to validate SNP associations.

**Response: Thank you for your time and expertise. The manuscript formatting and language have been thoroughly edited. A QQ plot has been added to the Results section.**

**Section 1.8 outlines the gene screening methodology.**

**“Structural data were obtained from the AlphaFold Protein Structure Database and the Protein Data Bank (PDB), with priority given to PDB entries. Selection criteria included the scientific name of the source organism (Homo sapiens), experimental method (X-ray diffraction or electron microscopy), and a refinement resolution of ≤2.5 Å.**

**A total of 17 mRNAs were identified through screening, among which only 3 possessed PDB IDs and contained small-molecule ligands. Based on the properties and size of the active sites, PYGL was ultimately selected for further investigation.**

Table 1. Structural data of the 17 mRNA-encoded proteins

| No. | Gene symbol | RNA type | PDB | | AlphaFold |
| --- | --- | --- | --- | --- | --- |
|  |  |  | PDB ID | Ligand ID |  |
| 1 | CDKN2B | mRNA |  |  | P42772 |
| 2 | BOC | mRNA | 3N1G | / |  |
| 3 | SCG5 | mRNA |  |  | P05408 |
| 4 | LRP1 | mRNA | 1D2L | / |  |
| 5 | ZCWPW2 | mRNA | 4Z0R | EDO: C_2_H_6_O_2_ |  |
| 6 | PYGL | mRNA | 3DDS | 26B: C_29_H_35_N_3_O_5_ |  |
| 7 | PREX1 | mRNA | 6VSK | / |  |
| 8 | SMAD7 | mRNA | 7CD1 | / |  |
| 9 | SLCO2A1 | mRNA |  |  | Q92959 |
| 10 | METRNL | mRNA |  |  | Q641Q3 |
| 11 | POU5F1B | mRNA |  |  | Q06416 |
| 12 | ABHD12B | mRNA |  |  | Q7Z5M8 |
| 13 | TCF7L2 | mRNA | 1JDH | / |  |
| 14 | CDH3 | mRNA | 5JYL | GOL: C_3_H_8_O_3_ |  |
| 15 | SOX6 | mRNA |  |  | P35712 |
| 16 | NKX2-3 | mRNA |  |  | Q8TAU0 |
| 17 | TMEM238L | mRNA |  |  | A6NJY4 |

Reviewer #4: The authors have successfully addressed all my previous comments and I don't have additional comments.

**Response: Thank you for your time and expertise.**

Reviewer #5: General Comments:
1. The manuscript initially mentions using three GWAS datasets but the revised version states that only one dataset, GCST90018808, was used. This change, supposedly in response to requests for replication and meta-analysis, is not a sound scientific solution. Removing datasets without clear, predefined criteria and without acknowledging the loss of statistical power and generalizability is problematic. The authors' response to Reviewers simply states that the single dataset was selected without explaining the rationale for discarding the others. A robust study would perform a meta-analysis to combine evidence from multiple cohorts or, at a minimum, demonstrate consistent findings (replication) across them. The arbitrary selection of a single dataset makes the findings highly susceptible to a single study's biases and reduces the ability to generalize results.
The authors claim to have filtered datasets based on sample size but only provide a non-specific cutoff of ncase < 5,000 as an example. The specific criteria used to select GCST90018808 over the others and what constituted a "small sample size" for the excluded datasets remain unclear in the text. This lack of transparency makes the data selection process appear arbitrary.

**Response: Thank you for your time and expertise.**

2. The authors mention the study population for the GWAS data but fail to provide the same demographic information for the RNA-Seq data, creating a data-context gap. This information is essential for interpreting the results and discussing generalizability.

A central claim of the study is the integration of GWAS and RNA-Seq data. However, the manuscript fails to explicitly address whether the populations in the GWAS and RNA-Seq datasets are matched. The GWAS data is from European and East Asian populations , while the RNA-Seq data is from the TCGA-COAD cohort. The TCGA is a US-based consortium, and its racial and ethnic composition may not align with the East Asian population that the authors repeatedly emphasize as a focus in their introduction and discussion. A failure to match populations can introduce significant confounding due to population stratification, undermining the validity of linking genetic variants to gene expression.

The authors mention using the TwoSampleMR R package and filtering for weak instrumental variables, but they explicitly state that they did not perform a Mendelian Randomization (MR) analysis. Furthermore, they fail to perform an eQTL analysis to directly link the identified SNPs to gene expression changes, which is a critical missing piece of the claimed "integrative analysis". Without this crucial link, the association between the selected genes and CRC is not robustly established, and the integration of GWAS and RNA-Seq data remains superficial and unconvincing.

**Response: Thank you for your time and expertise.**

3. The manuscript contains contradictory statements regarding data preprocessing. For RNA-Seq data, the method mentions filtering out genes and samples with more than 1% missing (NA) values, but the results section mentions excluding genes with more than 50% missing values. This is a significant discrepancy that raises serious questions about the rigor and reproducibility of the methodology.
The Abstract is poorly structured. It mixes methodological steps with results, making it difficult to follow the logical flow of the study. For instance, it lists specific numbers of identified mRNAs and lncRNAs in the "Method" section, which should be in the "Result" section. Similarly, the Conclusion section provides new examples of genes (CDKN2B, BOC, and METRNL) that are listed as potential therapeutic targets, which should be presented and discussed in the main result.

The manuscript lacks a concise and impactful conclusion. The concluding paragraphs are repetitive and contain general statements about the value of computational methods, rather than synthesizing the specific and unique contributions of this study.

**Response: Thank you for your time and expertise.**

**1% missing (NA) values：The missing value threshold stated as 1% in the original text should be corrected to 50%, which was applied as a filtering criterion during data normalization using log2(X+1) transformation.**

**50% missing values：For the Limma analysis, samples with zero expression values in >50% of genes were removed.**

**Both the Abstract and Conclusion sections have been revised.**

4. The authors acknowledge the lack of experimental validation but their responses and the manuscript's tone do not sufficiently temper the "overstated claims" of identifying "promising therapeutic targets". The claim that "the incorporation of extensive positive and control datasets enhances the robustness and reliability of the findings" is misleading, as this does not substitute for independent biological validation and is not adequately supported by the current work.

While survival analysis is a valuable component of the study, the authors do not specify if they performed a multiple testing correction for the survival analysis of the 18 screened genes. Given the number of tests performed (at least 18 genes x 3 survival metrics = 54 tests), uncorrected p-values (p < 0.05) are highly susceptible to false positives. This should be clearly stated and, if not performed, acknowledged as a limitation.

The GO and KEGG enrichment analyses are based on a very small gene list and, as the authors acknowledge in their response to Reviewer, often include only one or two genes per term. This provides minimal insight and weakens the biological interpretation of the findings. The conclusion that these analyses are meaningful and support the findings is not well-justified.

**Response: The specified content has been deleted as requested.“the incorporation of extensive positive and control datasets enhances the robustness and reliability of the findings”**

**The content has been added in Section 1.5.“The generated p value does not include correction for multiple hypothesis testing,”**

5. The GWAS Manhattan plot (Fig 2) shows a significance threshold of p<1e-6 , but the text also mentions a genome-wide significance threshold of p>5e−8, which is typically a much stricter standard. This inconsistency needs clarification.

**Response: While the conventional genome-wide significance threshold is typically set at p < 5e-8, we considered single nucleotide polymorphisms (SNPs) with p < 1e-6 as potentially significant in our study. Using this less stringent threshold (p <1e-6) for initial screening, we identified and validated several SNPs. Subsequent analysis revealed three genes CDKN2B, BOC, and METRNL showing significant associations, with p-values ranging between 5e-8 and 1e-6.**

Reviewer #6: The study attempts to find potent therapeutic targets in colorectal cancer by analyzing the genomic data of European and East Asian populations. The researchers suggested the PYGL gene as a potential therapeutic target, as it has interacting motives with the suggested therapeutic small molecules. All work was performed by a computation prediction application, which may facilitate finding targets and therapies for cancer and reduce the time of experimentation. The major limitation of this work is the lack of validation and experimentation, as the researcher confesses in their work.

**Response: Thank you for your time and expertise.**
